# The Neuroprotective Effect of the Association of Aquaporin-4/Glutamate Transporter-1 against Alzheimer's Disease

**DOI:** 10.1155/2016/4626593

**Published:** 2016-01-06

**Authors:** Yu-Long Lan, Shuang Zou, Jian-Jiao Chen, Jie Zhao, Shao Li

**Affiliations:** ^1^Department of Physiology, Dalian Medical University, Dalian 116044, China; ^2^Department of Neurosurgery, The First Affiliated Hospital of Dalian Medical University, Dalian 116011, China; ^3^Department of Neurosurgery, The Second Affiliated Hospital of Dalian Medical University, Dalian 116027, China; ^4^Liaoning Engineering Technology Centre of Target-Based Nature Products for Prevention and Treatment of Ageing-Related Neurodegeneration, Dalian 116044, China

## Abstract

Alzheimer's disease (AD) is a progressive neurodegenerative disorder that is characterized by memory loss and cognitive dysfunction. Aquaporin-4 (AQP4), which is primarily expressed in astrocytes, is the major water channel expressed in the central nervous system (CNS). This protein plays an important role in water and ion homeostasis in the normal brain and in various brain pathological conditions. Emerging evidence suggests that AQP4 deficiency impairs learning and memory and that this may be related to the expression of glutamate transporter-1 (GLT-1). Moreover, the colocalization of AQP4 and GLT-1 has long been studied in brain tissue; however, far less is known about the potential influence that the AQP4/GLT-1 complex may have on AD. Research on the functional interaction of AQP4 and GLT-1 has been demonstrated to be of great significance in the study of AD. Here, we review the interaction of AQP4 and GLT-1 in astrocytes, which might play a pivotal role in the regulation of distinct cellular responses that involve neuroprotection against AD. The association of AQP4 and GLT-1 could greatly supplement previous research regarding neuroprotection against AD.

## 1. Introduction

Aquaporin-4 (AQP4) is the predominant aquaporin expressed in the brain that provides the major route for water movement across plasma membranes [1]. Most of the studies that have been conducted on the physiological role of AQP4 have primarily focused on its contributions to water homeostasis and to certain pathophysiological processes such as ischemia, cerebral edema, epilepsy, K^+^ spatial buffering, and the development or integrity of the blood-brain barrier [2]. Therefore, AQP4 has been considered to be a critical modulator for both water and ion homeostasis in the brain. Recently, it has been reported that AQP4 plays a role in synaptic plasticity and that AQP4 deficiency impaired synaptic plasticity, spatial memory, and associative fear memory [3, 4]. However, little is known about the potential role of AQP4 in synaptic plasticity, especially in memory-associated areas in the brain such as the hippocampal CA1 region [5] and the parietal cortex [6].

Glutamate is the principal excitatory amino acid neurotransmitter in the mammalian brain. Glutamate-activated N-methyl-D-aspartate (NMDA) receptors increase Ca^2+^ influx into the cytosol, which triggers changes in neuronal metabolism and gene expression that are necessary for brain function. However, excessive release of glutamate could induce cell death through excitotoxicity, oxidative stress, or both, which leads to the development of neurofibrillary tangles, and, in turn, AD [7]. Therefore, the maintenance of a physiological level of extracellular glutamate is important for normal synaptic transmission and for the prevention of excitotoxicity. Clearance of glutamate from the extracellular space is accomplished primarily by transporter-mediated uptake of widely localized astrocytic glutamate transporters (GLTs) [8]. Within many brain regions, the dominant glutamate transporter is glutamate transporter-1 (GLT-1), while the glutamate/aspartate transporter (GLAST) is only highly expressed in the molecular layer of the cerebellum [9]. Previous studies have shown that glutamate uptake is reduced after A*β* infusion, which results in high extracellular glutamate concentrations, and, consequently, excitotoxicity [10, 11]. Conversely, enhancement of GLT-1 may be beneficial for the treatment of certain neurodegenerative disorders such as AD [12].

Glutamate is released into the synaptic cleft during neural activity, and water is actively transported by GLTs along with the reuptake of glutamate. In addition, the passive water permeability of GLTs is increased in the presence of glutamate and during depolarization of the cell [13]. It has been reported that one consequence of elevated glutamate levels is cell swelling, which in this case occurs primarily in astrocytes [14]. Furthermore, reports [15] have also suggested that astrocytic uptake of glutamate is important for the initiation of swelling. Together with the notion that AQP4 is the predominant water channel protein in the mammalian brain and is believed to be important for water homeostasis in the brain, the interaction between AQP4 and GLT-1 is thought to be an intimate spatial relationship [16–18]. It is believed that the finding of the colocalization of AQP4 and GLT-1 may be of great significance in terms of the treatment of various neurodegenerative disorders such as AD, and therefore, more significant research efforts should be directed toward this area.

## 2. The Localization of AQP4 and GLT-1 in Astrocytes

MacAulay et al. have provided a series of indications [13, 19] that, at the pial and vascular interfaces, the AQP4 pool is concentrated in the subpial and perivascular astrocytic endfeet. On the contrary, the water transport properties of the astroglial cell membrane that faces the neuropil are determined predominantly by GLTs. If this is true, GLTs may also impact the regulation of water balance, ionic balance, and various other agents in the synapse that are closely related to synaptic activity. Unfortunately, these two studies were performed in oocytes and not in intact brain tissue. Therefore, more efforts should be directed toward the clarification of the distinct distribution and physiological roles of AQP4 and GLTs for water homeostasis in the central nervous system. The coexpression of AQP4 and GLTs was discovered in the early 1990s by Nielsen et al. [20], who demonstrated that AQP4 is prominently expressed in astrocytes where GLTs are localized. Since then, a great deal of interest and excitement arose in the exploration of whether the parallel location of AQP4 and GLTs reflects a functional obligatory coupling. However, regrettably, the object of this study was GLAST. After that, additional evidence further revealed the close relationship between AQP4 and GLT-1, and it seems that the AQP4 and GLT-1 in the astrocyte cell membrane might exert effects on the nervous system as a functional complex [16–18]. Recent data further suggested that GLT-1 and AQP4 exist in astrocytic membranes as a macromolecular complex, as neuromyelitis optica-immunoglobulin G autoantibodies directed against AQP4 result in the concomitant loss of GLT-1 [16, 21]. Presently, using brain slices from individuals with ischemic stroke, Mogoanta et al. [22] conducted the first study on AQP4 and GLT-1 integration at the tissue level. They found that an increase in the colocalization of AQP4 and GLT-1 is a reaction to ischemia. They presented the idea that the application of the AQP4/GLT-1 regulator can ameliorate the therapeutic effects of stroke. With these coherent findings, an interesting hypothesis could be proposed in that there might be a correlation between the association of AQP4/GLT-1 and AD.

According to previous studies, AQP4 and GLT-1 might be concentrated in separate membrane domains. AQP4 is highly concentrated in those astrocytic membrane domains that face blood vessels and the pia, while GLT-1, in contrast, functions and is enriched in those astrocytic membrane domains that face the neuropil and synapses [20, 23]. However, this does not actually lead to the conclusion that it is impossible for GLT-1 to be coenriched with AQP4 in the perivascular space or in the subpial endfoot membranes where AQP4 is expressed in abundance. Intriguingly, the most recent study has offered an amendment to the ideas regarding the distribution of GLT-1. Schreiner et al. [24] demonstrated that, in neonatal and juvenile animals, discrete clusters of GLT-1 were also detected within the perivascular endfeet and that these clusters seem to preferentially colocalize with GFAP. Hence, the idea that the two molecules colocalize to form a supramolecular complex could not be denied simply because the prime functions of AQP4 and GLT-1 are assigned to different membrane domains. Moreover, it is known [20] that the perisynaptic region often features low densities of AQP4 and that the macromolecular AQP4/GLT-1 complexes at this site could actually exert the most important effects in anti-AD neuroprotection.

## 3. Synergistic Protective Effect of AQP4 and GLT-1 against Glutamate-Induced Neuronal Injury by A***β***

The regulatory potential of AQP4 on the function of GLT-1 with respect to synaptic plasticity and memory is clear. The finding of the physical interaction of AQP4 with GLT-1 suggests that this complex may be relevant for GLT-1 signaling mechanisms that are initiated within the neuronal membrane. In astrocytes, GLT-1 exists in a macromolecular complex with AQP4 [25], and in primary cultured astrocytes, both the expression of GLT-1 and glutamate uptake are downregulated by AQP4 deficiency [18]. These data indicate that AQP4 is not only coenriched with GLT-1, but could also regulate GLT-1 function. The study by Zeng et al. [18] provided the first direct evidence that AQP4 plays an important role in the function of GLTs. Through an analysis of cellular morphology, real time reverse transcriptase polymerase chain reaction (RT-PCR), and quantitative densitometry, they investigated the regulatory role of AQP4 in GLTs in primary cultured astrocytes from AQP4 knockout mice. They demonstrated that a lack of AQP4 downregulated astrocytic expression of GLT-1 but not that of GLAST [18]. Therefore, it is thought that the knockout of AQP4 might disturb the direct physical contact between AQP4 and GLT-1 as well as their signal transduction pathway and the functions of GLT-1. However, this might be just one part of the entire explanation. A direct consequence of AQP4 knockout, or merely a consequence of water and ion imbalances that result from an AQP4 knockout, may also explain GLT-1 downregulation. A previous study revealed that AQP4 deficiency could downregulate GLT-1 expression and glutamate uptake in cortical astrocytes [18]. Furthermore, the findings of Li et al. [4] implied that AQP4 plays a role in synaptic plasticity in the amygdala via the regulation of GLT-1 expression. Similarly, Yang et al. [17] found that upregulation of GLT-1 expression by Ceftriaxone (a *β*-lactam antibiotic), which is known to upregulate GLT-1 in astrocytes, can rescue AQP4 deficiency-induced impairment of synaptic plasticity [26]. This suggests that AQP4 functions as the modulator of synaptic plasticity and memory and that chronic GLT-1 upregulation rescues hippocampal memory deficits induced by the AQP4 knockout. Further studies are needed to clarify this correlation and to demonstrate why the decreased expression of AQP4 might induce a decrease in GLT-1.

It is well known that astrocytes play a significant role in synaptic plasticity, which is defined as the ability of neurons to change their synaptic strength [27]. Additionally, the A*β* cascade is crucial in the etiology of AD, as deficits in the clearance of A*β* from the brain parenchyma are considered to be potential contributors to the onset of sporadic AD [28]. Low levels of glutamate or endogenous synaptic activity may enhance dendritic spine growth [29, 30]. In contrast, excessive glutamate can precipitate the loss of dendrites and spines [29, 31]. Moreover, normal endogenous levels of A*β* may increase physiological synaptic glutamate release [32]. It has also been demonstrated that A*β* induces glutamate release from astrocytes, which in turn results in neuronal extracellular NMDAR (eNMDAR) activation; this then leads to both molecular and functional changes and thus heralds synaptic damage [33]. Glutamate translocation is a multistep process that moves glutamate against its concentration gradient and that utilizes energy stored in the Na^+^/K^+^ electrochemical gradient [34]. Conventionally, the direction of transport is inward under physiological conditions, but glutamate is transported in the outward direction in excitotoxic conditions when the extracellular [Na^+^]/intracellular [K^+^] ratio decreases and/or when the intracellular [Na^+^]/extracellular [K^+^] ratio increases [34]. Such GLT-1 reversal was also shown in ischemic glutamate release [35]. Thus, it remains to be clarified whether GLT-1 activators could facilitate glutamate clearance under excitotoxic conditions, or if this would instead intensify reverse transport [34], because GLT-1 reversal might lead to neuronal damage. Furthermore, the Na^+^/K^+^-ATPase that maintains these ion gradients is compromised in the injured brain [36, 37]. Therefore, the Na^+^/K^+^-ATPase may have important regulatory potential in glutamate transport under physiological conditions.

**Figure 1:**
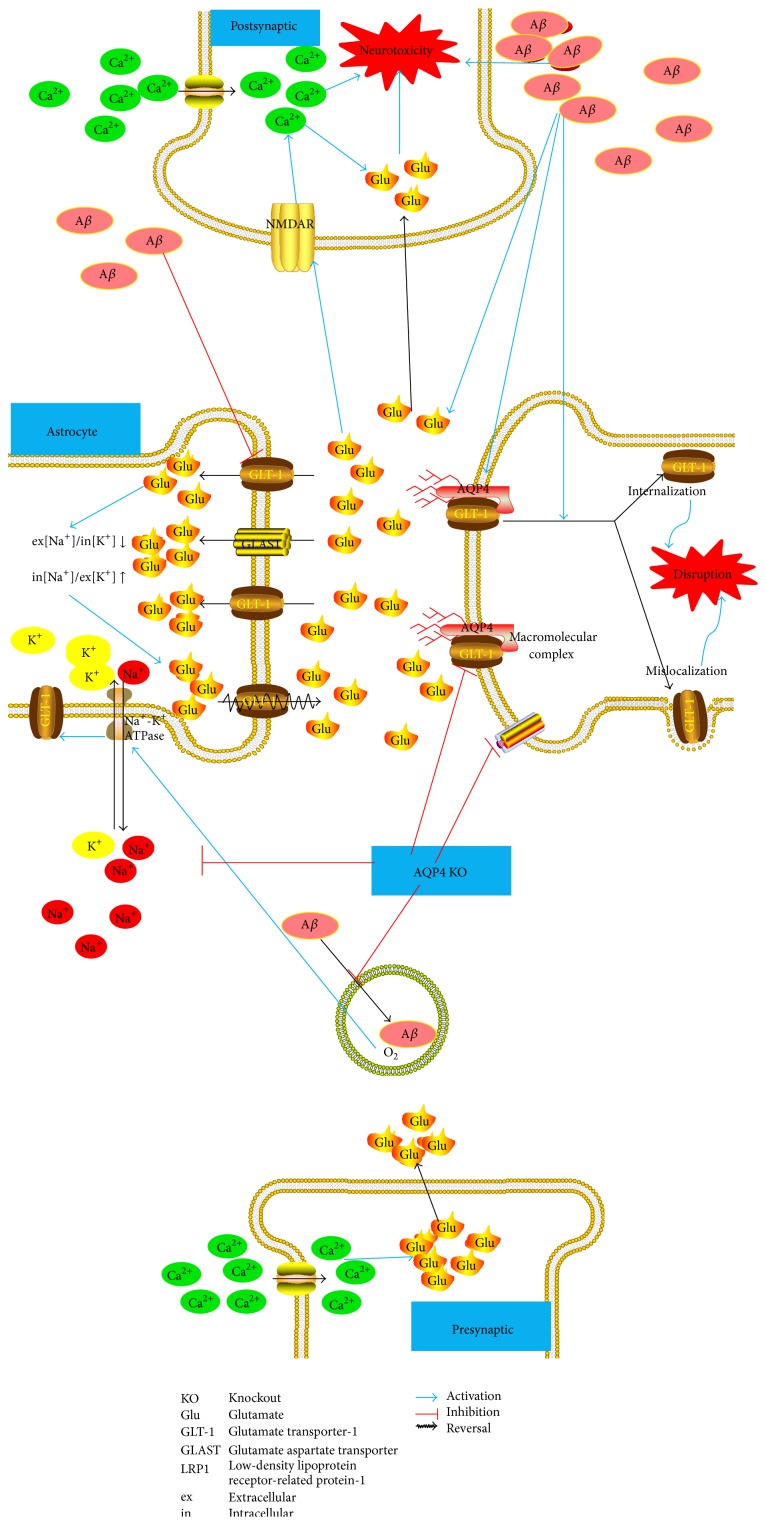
Neuroprotective effect of the association of AQP4/GLT-1 against glutamate-induced neuronal injury by A*β*. (1) A*β* targets. A*β* may increase the release of physiological synaptic glutamate. This in turn results in neuronal NMDAR activation, which leads to synaptic damage and neurotoxicity. Glutamate uptake by astrocytes is also reduced after A*β* infusion, which results in high extracellular glutamate concentrations, and, consequently, excitotoxicity. Glutamate dyshomeostasis could play a role in the pathogenesis of AD, and in this process, GLT-1 has demonstrated to undergo oxidative damage by exposure to A*β*. (2) Glutamatergic system. GLT-1 is the major glutamate transporter that is responsible for various essential neuroprotective functions that include the prevention of glutamate-mediated injury of neurons and synapses; it accomplishes this through the transport of glutamate in the inward direction under normal conditions. In excitotoxic conditions, when the extracellular [Na^+^]/intracellular [K^+^] ratio is decreased and/or when the intracellular [Na^+^]/extracellular [K^+^] ratio is increased, glutamate is transported in the outward direction, such that GLT-1 reversal might further intensify glutamate-induced synaptic damage. (3) Consequences of AQP4 knockout. It is known that AQP4 deficiency may downregulate both GLT-1 expression and glutamate uptake, but it may also downregulate LRP1, which is involved in A*β* clearance. Moreover, AQP4 deficiency could cause the inhibition of oxygen delivery from brain microvessels to the Na^+^/K^+^-ATPase. This might represent another important factor in the regulation of Na^+^/K^+^-ATPase function and ultimately the neuroprotection of GLT-1 against AD toxicity. (4) Neuroprotective effects of the association of AQP4 and GLT-1. As a functional complex, AQP4 and GLT-1 in astrocyte cell membranes could exert neuroprotective effects, and the mislocalization and internalization of GLT-1 in astrocyte membranes, as promoted by A*β*, might be responsible for the disruption of this macromolecular complex. This leads to a marked reduction in the rate at which astrocytes clear glutamate from the extracellular space, and, most importantly, to the development of AD.

Like GLT-1, AQP4 in the brain is involved in synaptic plasticity [38] and may also play important roles in antineurotoxicity. Intriguingly, in addition to the demonstration that AQP4 deletion increases NADH fluorescence in areas furthest away from cerebral microvessels, the study conducted by Thrane et al. [39] provided the first line of evidence that AQP4 impacts the oxygenation of brain tissue. This study suggests that K^+^ uptake is suppressed in AQP4 knockout mice as a consequence of decreased oxygen delivery to tissue that is located the furthest from a vascular oxygen source. This finding might expedite research regarding neuroprotection against AD toxicity via the regulation of ion imbalance by AQP4. Thus, the inhibition of oxygen delivery in the setting of AQP4 deficiency might represent another important factor in the regulation of the Na^+^/K^+^-ATPase and ultimately the neuroprotection by GLT-1 against AD toxicity. Low-density lipoprotein receptor-related protein-1 (LRP1) is expressed in the perivascular endfeet of astrocytes and in brain microvascular endothelial cells; it mediates a continuous efflux of brain A*β* into the circulation [40]. Increased LRP1 expression has been identified in fine astrocyte processes that surround senile plaques (SPs) (AD is characterized pathologically by abnormal accumulation of extracellular aggregates of A*β* in the form of SPs [41], and a major component of these SPs is A*β*_1–42_) [42]. LRP1 has also been shown to be necessary for A*β*-induced astrocyte activation [43], and thus it can be concluded that LRP1 is involved in A*β* clearance, which is mediated by activated astrocytes both in vivo and in vitro [44]. Yang et al. [44] demonstrated that AQP4 deficiency decreases LRP1 upregulation and A*β* uptake, which reduces astrocyte activity. Therefore, AQP4 may be significant in the upregulation of LRP1 and in the clearance of A*β*. AQP4 and GLT-1 are speculated to share an intimate spatial relationship and to be a part of the same supramolecular complex. It has been demonstrated that A*β*_1–42_ in AD induces rapid GLT-1 mislocalization and internalization in astrocytes. This results in a marked reduction in the rate at which astrocytes clear synaptically released glutamate from the extracellular space, which inhibits the functions of GLT-1 [45]. Moreover, the disruption of this macromolecular complex might be partly attributed to GLT-1 mislocalization and internalization in astrocytes (Figure 1). Although further clarification is still needed, various recent studies have shown functional interactions of AQP4 and GLT-1; more efforts should be directed toward the elucidation of the physiological relevance of this complex to help clarify the promising neuroprotective effects of these proteins against AD. Most importantly, the hypothesis may be put forth that the interaction of AQP4 and GLT-1 in astrocytes may play a pivotal role in the regulation of distinct cellular responses that are directed toward neuronal preservation and neuroprotection against AD.

## 4. The Disruption of the Association of AQP4/GLT-1 in Alzheimer's Disease

Results obtained from studies that have included AQP4 knockout astrocytes have provided direct evidence for the interaction of AQP4 and GLT-1 [18]. Additionally, the interaction between AQP4 and GLT-1 is thought to be an intimate spatial relationship, where these two proteins are part of the same supramolecular complex [16–18]. Despite this evidence, an in-depth morphometric characterization of the relationship of GLT-1 with AQP4 with respect to the pathology of AD is still lacking.

The study conducted by Yang et al. [46] showed a significant increase in AQP4 expression in AD mice at the age of 9 months compared with wild type according to SDS-Page and western blot. The tg-ArcSwe mouse model of Alzheimer's disease was used in this study, as this model displays perivascular plaques as well as plaques that are confined to the neuropil. However, many studies have found that GLT-1 is significantly reduced or damaged in AD; more recent studies have further confirmed that both the GLT-1 mRNA and protein levels are reduced in AD [47–52] according to RT-PCR and western blot experiments, respectively. In addition because GLT-1 undergoes oxidative damage by exposure to A*β* [53–57], this raises the possibility that glutamate dyshomeostasis plays a role in the pathogenesis of AD. Intriguingly, contradictory results have been obtained in regard to GLT-1 expression in AD mice, which still need to be clarified. Previously, in the late 1990s, Beckstrøm and colleagues in their elegant study of AD patients aged 69 to 94 [58] observed individual differences in the levels of glutamate transporters. Therefore, this provided a rejection of a straightforward correlation between reduced glutamate transporter expression and AD [59]. Because this study was performed in human tissues, their results might be more convincing. Furthermore, a more recent study performed by Kulijewicz-Nawrot et al. also indicated that astrocytic GLT-1 expression in 3xTg-AD mice showed no significant difference at any age when they were compared with control animals [60]; these data were obtained by histograms and representative western blots. The authors found GLT-1 expression in the mPFC to be generally unchanged, which might suggest the preservation of glutamate uptake or possible differences in transporter expression between subjects [60]. Their results were in line with Beckstrøm et al.'s findings, which highlight the variability in transporter expression. These contradictory results may be due to different methodologies and techniques used. Moreover, it was previously noted that A*β*_1–42_ in AD induces rapid internalization and mislocalization of GLT-1 in astrocytes, which results in a marked reduction in the rate at which astrocytes clear synaptically released glutamate from the extracellular space [45]. Thus, it is thought that a disruption of the association of AQP4/GLT-1 might be partly attributed to GLT-1 mislocalization or internalization. This combined with the loss of GLT-1 in AD brains as well as in animal models [47, 51, 52] may be observed in neuronal membranes in AD. This may be of great functional significance to the neuroprotective effect of AQP4 and GLT-1, as this contributes to neuronal impairment. Furthermore, glutamate transporter variants may also be responsible for the reduced glutamate uptake and thus the neurotoxicity in AD [49]. All of these factors might cause the disruption of the AQP4/GLT-1 association in AD.

## 5. Conclusion and Future Studies

There is a general agreement that AQP4 may exert neuroprotective effects against A*β* toxicity through its binding to GLT-1 at the plasma membrane. In addition, this water channel has been shown to contribute to GLT-1 function. Because it is plausible that the involvement of AQP4 in the modulation of A*β* toxicity might be related to channel activation, then porin interactions with glutamate-activated GLT-1 may be crucial in the maintenance of AQP4 activation. Increasing evidence suggests that AQP4 and GLT-1 in the plasma membrane might be designated as dynamic signaling platforms; the interaction of these two proteins has a potential role in the regulation of distinct cellular responses that are directed toward neuronal preservation and neuroprotection against AD. It is enticing to speculate that a disruption of the AQP4/GLT-1 association, which has been observed in neuronal membranes in AD brains, may contribute to neuronal impairment. Furthermore, various studies have detailed the interaction of AQP4 with other signaling proteins that are involved in neuronal maintenance. Along these lines, the association of AQP4/GLT-1 with other proteins such as Kir4.1 [61, 62] may represent a relevant macromolecular complex in the plasma membrane. Thus, further explorations of these potential porin modifications at the neuronal membrane as a consequence of protein rearrangements in the plasma membrane might be a worthy pursuit [63]. This would provide a better understanding of the consequences of impaired protein associations that are related to AD.
